# The efficacy of unicondylar knee arthroplasty for medial compartment arthritis of the knee combined with anterior cruciate ligament dysfunction

**DOI:** 10.1186/s12893-024-02482-4

**Published:** 2024-06-17

**Authors:** Yonghui Qin, Jia Li, Guoxing Jia, Jun Li, Zhenshuan Zhao, Xiaoguang Yu

**Affiliations:** Department of Orthopedic Surgery, The First Hospital of Hebei Medical University, Shijiazhuang, China

**Keywords:** UKA, OA, ACL, KOOS, Function

## Abstract

**Background:**

To investigate the outcome and prognosis after Unicondylar knee arthroplasty (UKA) in patients with medial compartment arthritis of the knee combined with anterior cruciate ligament(ACL) dysfunction.

**Methods:**

A total of 122 patients diagnosed with knee osteoarthritis and treated with medial mobile platform unicondylar replacement at our center from January 2019 to December 2021 were retrospectively included in the study, and were divided into two groups according to ACL function, namely the normal ACL function group (ACLF) and the poor ACL function (N-ACLF) group. The postoperative results and prognosis of the two groups were evaluated and compared.

**Results:**

This study included 122 patients who underwent UKA surgery. There were no statistical differences in preoperative and postoperative posterior tibial tilt angle, knee mobility, KOOS, and prognosis between the two groups (*P* > 0.05).

**Conclusion:**

For medial compartment arthritis of the knee combined with ACL malfunction, surgery resulted in pain relief, improved quality of life and a good prognosis for such patients. It is hoped that clinicians will perform UKA in patients with ACL dysfunction after a comprehensive evaluation to improve their quality of life.

## Introduction

Unicondylar knee arthroplasty (UKA) is a bone-preserving and ligament-preserving procedure that reliably restores normal knee kinematics and function in arthritic joints limited to the medial or lateral side of the knee [1].The UKA technique was introduced in clinical practice in the late 1960s and 1970s, the first of which was the St Georg Sled prosthesis designed by Buchholz, which was 1969 when it was first used. Clinical studies on these prostheses showed excellent results, and Engelbrecht et al. found that 85% of the 226 implanted prostheses were pain-free [2].MacKinnon later found good improvement in knee function in 79% of patients [3]. The Oxford unicondylar replacement system, designed and developed by Professor John Goodfe11ow and Professor John O’Connor of the University of Oxford and the Royal College of Surgeons, was first used clinically in the United Kingdom in 1976 [4]. The history of decades of clinical use has amply demonstrated its excellent reliability. Its 10-year prosthesis survival rate of 94% and 20-year follow-up results of 91% were reported in the UK. The Oxford unicondylar replacement was the first movable platform knee unicondylar replacement system approved by the US FDA. The Oxford UKA heralded the greatest advancement in modern UKA.

Anterior cruciate ligament (ACL) injuries of the knee are a common clinical condition, with Johnson DL et al [5] reporting an annual incidence of over 250,000 ACL tears. Arthroscopic ACL reconstruction is a better way to treat simple ACL injuries, and patients can achieve faster and better functional recovery. However, osteoarthritis (OA) of the knee combined with ACL deficiency is more common clinically, and the interrelationship between the two and the mechanism of their occurrence has been more controversial. There are two main views, namely primary knee OA secondary to ACL injury and primary ACL injury secondary to knee OA. This study focuses on the former, in which the ACL is generally intact at the early stage of the lesion and is dominated by wear of the anterior medial cartilage of the tibial plateau [6]. As degeneration increases, the ACL is subjected to wear and tear of the surrounding bones and secondary rupture occurs, while wear and tear of the tibial plateau progresses further posteriorly with the combination of medial collateral ligament contracture and lateral compartment OA.

The primary role of the ACL is to limit excessive movement of the tibia anteriorly to maintain the knee within its normal range of motion. ACL injury alone increases the risk of developing OA tenfold [7]. Goodfellow found a 21.4% revision rate for Oxford UKA over two years in ACL-deficient knees [8]. However, in contrast, Boissonneault et al. showed no difference in prognosis between ACL-intact and ACL-deficient knees at 5 years after UKA, with a posterior tibial slope of 4.7° preoperatively and 2.5° postoperatively in the ACL-deficient group [9]. There is no consensus on the treatment for such patients. Data from studies of such patients in China are scarce.Therefore, this study investigated the outcome and prognosis after UKA in patients with medial compartment arthritis of the knee combined with anterior cruciate ligament dysfunction (synovial damage, longitudinal splits, friable and fragmented) to provide some theoretical basis for the treatment of this condition with UKA.

## Materials and methods

### Patient selection

Total of 122 patients diagnosed with knee osteoarthritis and treated with medial mobile platform unicondylar arthroplasty performed at our center from January 2019 to December 2021 were included in this retrospective study, and were divided into two groups according to ACL function, namely the normal ACL function group (ACLF) and the ACL dysfunction (N-ACLF) group(synovial damage, longitudinal splits, friable and fragmented). Inclusion criteria: (a) patients with medial knee OA who met the indications for UKA [10]; (b) application of Oxford artificial unicondylar joint replacement prosthesis for medial UKA, and for patients in the ACL dysfunction group, UKA was performed along with cleaning of the bone fragments affecting the trajectory. Exclusion criteria: (a) ACL status was not recorded; (b) Fixed flexion deformity angle less than 15°; (c) follow-up period was less than 24 months. All patients underwent routine preoperative the full length weight-bearing position x-ray, lateral x ray, axial x ray of the knee joint, Varus and valgus stress radiographs and underwent knee joint CT and MRI examination. The studies involving human participants were reviewed and approved by Ethics Committee of The First Hospital of Hebei Medical University(Z2021-003-1).

### ACL functional assessment

During the procedure, the surgeon assesses the function of the ACL by visual inspection and by pulling on the ACL with a tendon hook. The ACL is then categorized as functioning and dysfunctioning (including: synovial damage, longitudinal fracture, fragility or fragmentation). The reasons for using the ACL assessment method are as follows: in knee osteoarthritis, changes in the size of tibial erosion or the presence of visually visible bone deformities, bone redundancy, and soft tissue contractures make it very difficult to accurately assess the functionality of the ACL by routine clinical examination. In addition, the presence of intercondylar notch bone redundancy can obscure the imaging of ACL on MRI. Therefore, the degree of ACL injury was evaluated using the anterior drawer test under preoperative anesthesia and the use of tendon probing hooks to pull the ACL during surgery in this study.

### Clinical evaluation

**Fig. 1:**
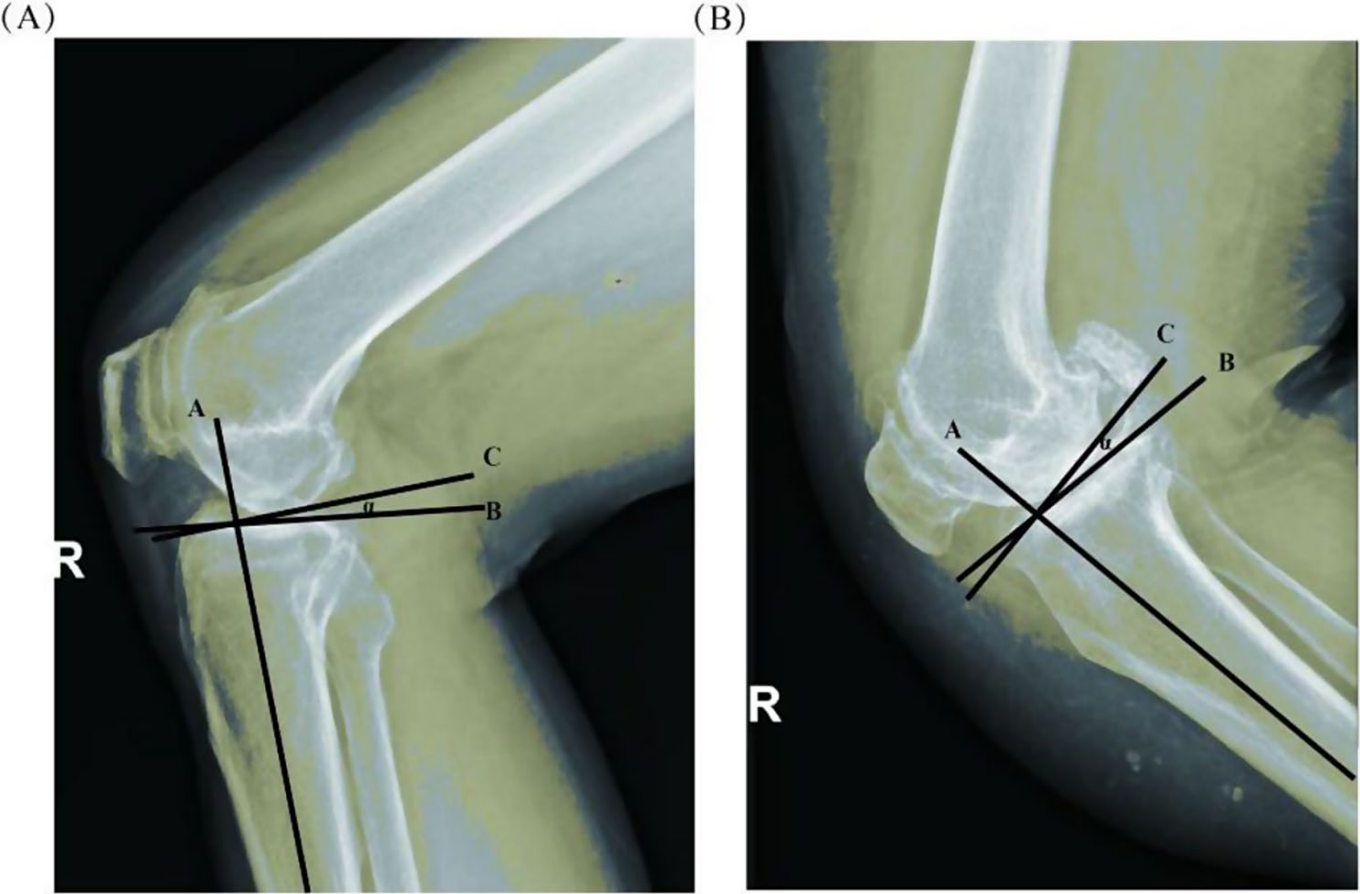
Measurement of the posterior tibial slope. Line A is the anatomical axis of the proximal tibial shaft, line B is the tangent of the tibial plateau, and Line C is the vertical line of Line A. Angle alpha is the posterior tibial slope. (**A**) Representative diagram of less femoropolar involvement; (**B**) Representative diagram of more femoropolar involvement

General clinical data including gender, age, BMI, and duration of disease were collected from the enrolled patients and followed up preoperatively and postoperatively. The follow-up included: measurement of posterior tibial slope (PTS) (Fig. 1), knee range of motion in extension and flexion, knee injury and osteoarthritis outcome score (KOOS) [11]; the presence of complications such as prosthesis loosening, infection, wear and tear, and dislocation were assessed.

### Statistical analysis

Data were expressed as mean ± standard deviation (SD). Two-group comparisons were performed using the independent samples *t-test*. *P* < 0.05 was considered statistically significant. Statistical analyses were performed using SPSS version 20.0 software (Chicago, IL, USA). GraphPad Prism 6.0 software was used for graphing the results.

## Results

### General information of patients

**Fig. 2:**
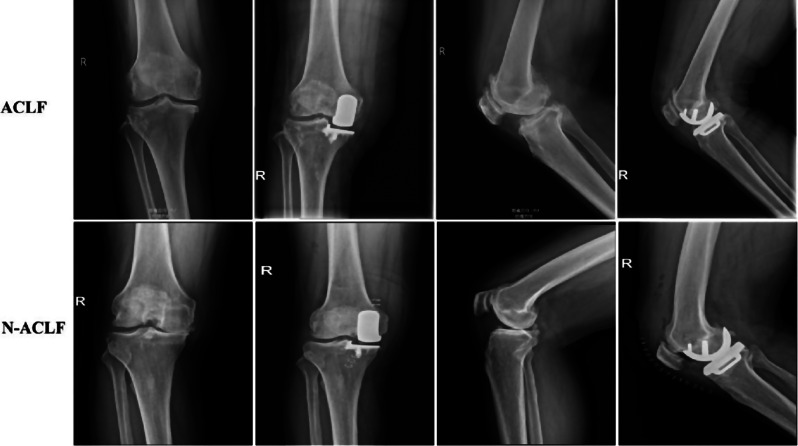
Representative imaging data of ACLF and n-ACLF groups

The study included 122 patients who underwent UKA, representative imaging data for both groups are shown in Fig. 2. The mean age, gender, BMI and duration of disease were not statistically different between the two groups (*P* > 0.05) (Table 1). In addition, 27 cases in the N-ACLF group had combined synovial damage, 19 cases had combined longitudinal splits, and 6 cases had partial or near-complete ACL tears.

**Table 1:** Demographic data

	ACLF group **(** ***n*** ** = 70)**	*N*-ACLF group **(** ***n*** ** = 52)**	***P*** **value**
Age(y)	62.0 ± 5.4	64.1 ± 4.8	0.054
Gender(M/F)	18/42	15/45	0.398
BMI(kg/m^2^)	26.8 ± 4.1	27.3 ± 3.7	0.478
Course of disease(y)	8.4 ± 2.1	9.1 ± 1.8	0.197

### Posterior tibial slope and knee flexion range of motion

Within-group comparisons showed that the posterior tibial slope was significantly reduced in the N-ACLF group compared with that before surgery (*P* < 0.05), while knee flexion range of motion was significantly improved in the ACLF and N-ACLF groups compared with that before surgery (*P* < 0.05). There was no statistical difference between the two groups in the preoperative and postoperative posterior tibial slope and knee flexion range of motion (*P* > 0.05) (Table 2).

**Table 2:** Comparison of posterior tibial slope and knee flexion range of motion

	ACLF group **(** ***n*** ** = 70)**	*P* value	*N*-ACLF group **(** ***n*** ** = 52)**	*P* value
Posterior tibial slope, °				
Preoperative	5.3 ± 3.5	—	7.2 ± 2.9*	0.021
Postoperative	5.1 ± 2.4	0.387	5.4 ± 2.6^#^	0.015
Flexion, °				
Preoperative	115.4 ± 23.1	—	119.5 ± 17.4	0.650
Postoperative	130.2 ± 18.5^#^	0.025	132.6 ± 15.7^#^	0.029

^*^P < 0.05 vs. ACLF group, ^#^*P* < 0.05 vs. preoperative

### KOOS scores

The KOOS Pain, KOOS Symptom, KOOS Activities of Daily Living, KOOS Sport and KOOS Quality of Life scores of patients in the ACLF and N-ACLF groups increased compared with the preoperative period, and all scores except KOOS Symptom were significantly (*P* < 0.05). There was no statistical difference between the ACLF and N-ACLF groups(Table 3).

**Table 3:** Comparison of knee injury and osteoarthritis outcomes score

	Preoperative assessment					6-months assessment			
	**ACLF group** **(** ***n*** **= 70)**		**N- ACLF group** **(** ***n*** **= 52)**			**ACLF group** **(** ***n*** **= 70)**	***P*** **value**	**N-ACLF group** **(** ***n*** **= 52)**	***P*** **value**
KOOS Pain	59.3 ± 12.4		61.2 ± 14.1			80.4 ± 13.3^#^	0.035	82.2 ± 16.1^#^	0.012
KOOS Symptom	60.5 ± 14.3		58.7 ± 15.2			63.8 ± 12.4	0.542	62.3 ± 13.1	0.117
KOOS Activities of Daily Living	61.5 ± 16.3		60.7 ± 14.2			76.5 ± 16.3^#^	0.023	79.6 ± 19.4^#^	0.018
KOOS Sport	40.5 ± 12.3		38.9 ± 15.1			55.4 ± 16.2^#^	0.013	59.7 ± 14.8^#^	0.002
KOOS Quality of Life	45.3 ± 10.2		41.6 ± 15.3			73.6 ± 21.3^#^	0.041	77.3 ± 18.4^#^	0.026
	**1-year Assessment**					**2-years Assessment**			
	**ACLF group** **(** ***n*** **= 70)**	***P*** **value**	**N-ACLF group** **(** ***n*** **= 52)**	***P*** **value**		**ACLF group** **(** ***n*** **= 70)**	***P*** **value**	**N-ACLF group** **(** ***n*** **= 52)**	***P*** **value**
KOOS Pain	86.6 ± 16.2^#^	0.047	87.1 ± 15.3^#^	0.021		89.9 ± 13.6^#^	0.029	90.3 ± 17.2^#^	0.017
KOOS Symptom	65.8 ± 17.2^#^	0.016	67.3 ± 18.5^#^	0.039		68.7 ± 15.3^#^	0.046	70.2 ± 16.4^#^	0.025
KOOS Activities of Daily Living	78.4 ± 15.3^#^	0.024	78.9 ± 18.4^#^	0.013		79.5 ± 17.2^#^	0.032	80.1 ± 16.5^#^	0.029
KOOS Sport	62.2 ± 15.6^#^	0.044	63.6 ± 13.9^#^	0.011		63.1 ± 17.4^#^	0.012	66.3 ± 12.8^#^	0.037
KOOS Quality of Life	75.6 ± 19.8^#^	0.032	78.3 ± 23.5^#^	0.018		79.4 ± 23.1^#^	0.041	80.3 ± 26.4^#^	0.015

^#^*P* < 0.05 vs. preoperative.

### Prognosis

During the two-year follow-up, none of the ACLF and N-ACLF groups required revision, and no complications such as prosthesis loosening, malposition, dislocation, or infection, fat embolism, or deep vein thrombosis were observed. All patients had good postoperative knee function and improved quality of life.

## Discussion

The primary function of the ACL is to prevent anterior dislocation of the tibial plateau and, to a lesser extent, to improve stability during rotation of the tibial plateau. The pathological classification of the ACL is mainly normal, with synovial damage, longitudinal splitting, fragmentation, and loss, and the extent of ACL damage is assessed by intraoperative visual observation while pulling the ACL with a tendon probe hook. The main reason for this approach is that the tibia is worn and deformed in patients with osteoarthritis of the knee, and the bone fragments and soft tissue contractures make it difficult to accurately assess the integrity of the ACL by traditional clinical physical examination methods. In addition, the bone fragments in the intercondylar fossa obstruct the normal image of the ACL in MRI, thus affecting the accuracy of MRI in diagnosing ACL injury [12].

Anteromedial osteoarthritis of the ACL-injured knee is a contraindication to unicondylar arthroplasty primarily because ACL deficiency predisposes to aseptic loosening of the tibial prosthesis after unicondylar replacement and accelerated eccentric wear of the spacer, thereby increasing the failure rate. In addition, without the protective effect of the ACL, wear of the tibial plateau extends posteriorly, causing posterior medial osteoarthritis, creating a fixed inversion deformity and accelerating the degeneration of the lateral intertrochanteric compartment [13].

Treatment of medial compartment OA combined with ACL defects in the knee is complex and controversial. The posterior tibial slope is important in unicondylar replacements for anteromedial osteoarthritis of the knee with ACL injury. Many studies have shown that maintaining proper tension in the medial collateral ligament is essential to ensure a good surgical outcome. Changing the tibial slope angle controls the appropriate tension of the medial collateral ligament; increasing the tilt angle decreases the tension of the medial collateral ligament and decreasing the slope increases the tension of the medial collateral ligament. Unicondylar replacement with fixed spacers requires a very small posterior tibial slope, which should be<7° [14]. Hernigou et al [15] showed that the revision rate of unicondylar replacement was not related to ACL injury, but rather to the posterior tilt angle of the tibial prosthesis. For every 10° increase in the posterior tibial slope, the tibial plateau will be displaced anteriorly by 6 mm during unicondylar stance, so it is recommended that the posterior tibial prosthesis should be < 7°. Jin C et al. [13] concluded that removal of the bone fragments causing intercondylar impingement is necessary to ensure good knee mobility after unicondylar arthroplasty, as is preservation of all ACL remnants to prevent postoperative instability.

Adaptive changes in the knee capsule are a common manifestation of chronic OA and usually include the appearance of contractures, bone fragments and scar tissue, the above tissues providing some stability to the knee joint in the absence of ACL. Marshall et al. found that in the absence of ACL, increased OA and thickening of the capsule, bone fragments developed along the border of the femoral condyle and as the capsule thickness increased, knee instability in the anterior-posterior plane decreased [16]. In the article by Brage et al. describing the loss of anterior-posterior laxity in the knee joint with unicompartmental OA, they hypothesized that the resulting bone flab production and soft tissue contracture may be a compensatory mechanism and lead to a decrease in laxity [17].

However some authors have questioned the need for an intact ACL when performing medial UKA. Engh et al. reported a survival rate of 94% at 6 years for ACL-deficient, fixed-bearing UKA and 93% for fixed-bearing UKA in knees with an intact ACL [18]. Tinius et al. reported interim follow-up results in 27 cases based on a previous study, with a follow-up time of 53 months, remained free of any complications and functioned well [19]. One of the most common complications in UKA with a mobile bearing is polymorphic location. Fixed bearing can avoid this complication. Previous studies have shown that fixed bearing can be used for patients with ACL defects and have a good prognosis, but the prerequisite for choosing a fixed platform is rotational stability, and the wear rate of fixed bearing is higher under the same material conditions [20, 21]. Although the follow-up time in the present study was only 2 years, the results of the present study were consistent with the above, i.e., there was no significant difference in tibial anterior displacement, range of motion, KOOS and prognosis of the knee after unicondylar replacement for ACL injury compared to the knee with good ACL function after reduction of the posterior tibial tilt angle.

The limitations of this study are the small number of patients and the short follow-up period. medial UKA of the ACL-deficient knee is a technically demanding procedure with a high learning curve. Therefore, caution should be exercised when performing this procedure, and technical errors such as failure to remove bone fragments, overfilling the joint, and causing collateral ligament imbalance should be avoided in this type of surgery.

## Conclusion

This is a study evaluating the outcomes of non-robotic, mobile bearing UKA. In ACL dysfunctional knees, the procedure resulted in pain relief, improved quality of life, and a favorable prognosis for such patients. The outcomes in the ACL dysfunctional group were not significantly different in all outcomes compared with UKA in ACL intact knees. We therefore hope that UKA will be performed in patients with ACL dysfunction after a comprehensive evaluation by clinicians to improve their quality of life.

## Data Availability

The raw data supporting the conclusions of this article will be made available by the corresponding author(13703212070@163.com), without undue reservation.

## References

[1] Johal S, Nakano N, Baxter M (2018). Unicompartmental knee arthroplasty: the past, current controversies, and future perspectives. J Knee Surg.

[2] Engelbrecht E, Siegel A, Rottger J et al. Statistics of total knee replacement: partial and total knee replacement, design St. Georg: a review of a 4-year observation. Clin Orthop Relat Res. 1976;(120):54–64. 975667

[3] Mackinnon J, Young S, Baily RA (1988). The St Georg Sledge for unicompartmental replacement of the knee. A prospective study of 115 cases. J Bone Joint Surg Br Vol.

[4] Goodfellow J, O’Connor J (1978). The mechanics of the knee and prosthesis design. J Bone Joint Surg Br Vol.

[5] Johnson DL, Warner JJ (1993). Diagnosis for anterior cruciate ligament surgery. Clin Sports Med.

[6] Annapareddy A, Mulpur P, Prakash M (2023). Partial versus total knee arthroplasty for isolated antero-medial osteoarthritis - an analysis of PROMs and satisfaction. SICOT J.

[7] riban JB, Vincent HK, Trojian TH (2023). Preventing Osteoarthritis after an Anterior Cruciate Ligament Injury: an Osteoarthritis Action Alliance Consensus Statement. J Athl Train.

[8] Goodfellow J, O’Connor J. The anterior cruciate ligament in knee arthroplasty. A risk-factor with unconstrained meniscal prostheses. Clin Orthop Relat Res. 1992;(276):245–52. 1537161

[9] Boissonneault A, Pandit H, Pegg E (2013). No difference in survivorship after unicompartmental knee arthroplasty with or without an intact anterior cruciate ligament. Knee Surg Sports Traumatol Arthrosc.

[10] Kang SN, Smith TO, De Sprenger WB (2011). Preoperative patellofemoral degenerative changes do not affect the outcome after medial Oxford unicompartmental knee replacement: a report from an independent centre. J Bone Joint Surg Br.

[11] Lysholm J, Gillquist J (1982). Evaluation of knee ligament surgery results with special emphasis on use of a scoring scale. Am J Sports Med.

[12] Wang H, Fang C, Tao M (2022). Hourglass-shaped grafts are superior to conventional grafts for restoring knee stability and graft force at knee flexion angle of 30° following anterior cruciate ligament reconstruction: a finite element analysis. Front Bioeng Biotechnol.

[13] Jin C, Song EK, Jin QH (2018). Outcomes of simultaneous high tibial osteotomy and anterior cruciate ligament reconstruction in anterior cruciate ligament deficient knee with osteoarthritis. BMC Musculoskelet Disord.

[14] Voos JE, Suero EM, Citak M (2012). Effect of tibial slope on the stability of the anterior cruciate ligament-deficient knee. Knee Surg Sports Traumatol Arthrosc.

[15] Hernigou P, Deschamps G (2004). Posterior slope of the tibial implant and the outcome of unicompartmental knee arthroplasty. J Bone Joint Surg(Am).

[16] Marshall JL, Olsson SE (1971). Instability of the knee. A long- term experimental study in dogs. J Bone Joint Surg Am.

[17] Brage ME, Draganich LF, Pottenger LA (1994). Knee laxity in symptomatic osteoarthritis. Clin Orthop Relat Res.

[18] Engh GA, Ammeen DJ (2014). Unicondylar arthroplasty in knees with deficient anterior cruciate ligaments. Clin Orthop Relat Res.

[19] Tinius M, Hepp P, Becker R (2012). Combined unicompartmental knee arthroplasty and anterior cruciate ligament reconstruction. Knee Surg Sports Traumatol Arthrosc.

[20] Zumbrunn T, Schütz P, von Knoch F (2020). Medial unicompartmental knee arthroplasty in ACL-deficient knees is a viable treatment option: in vivo kinematic evaluation using a moving fluoroscope. Knee Surg Sports Traumatol Arthrosc.

[21] Plancher KD, Briggs KK, Brite JE (2022). Dorr Surgical Techniques & Technologies Award: patient acceptable symptom state (PASS) in medial and lateral unicompartmental knee arthroplasty: does the Status of the ACL impact outcomes?. J Arthroplasty.

